# Supplementary material for: Patterns of Intron Gain and Loss in Fungi
Source: PLoS Biol. 2004 Nov 30;2(12):e422. doi: 10.1371/journal.pbio.0020422 (PMC532390; doi:10.1371/journal.pbio.0020422)
Supplement: Table S1 — Also available at http://genes.mit.edu/NielsenEtAl/. (4.3 MB ZIP). [file pbio.0020422.st001.zip › NielsenEtAl/html/717.html]

AN8534.1.NCU01882.1.MG08279.1.FG11617.1


```
 CLUSTAL W (1.82) Multiple Sequence Alignments - Introns Inserted


Sequence 1: NCU01882.1	594 aa
Sequence 2: FG11617.1	584 aa
Sequence 3: MG08279.1	615 aa
Sequence 4: AN8534.1	580 aa
Alignment Length: 622 aa
Number Identitical Residues: 297 aa
Alignment Score (without introns) 14976


MG08279.1 	MG----WVQKIDHAIGRSVVGRWFQLDGSGH~HKERKGSNFSTEIRAGLATFFAMS2YII
NCU01882.1	MGKVGDWVDTVNRKVAASKVGYWFKLDGSGH0PKERKGSYFFTEMRAGLATFFAMS2YII
FG11617.1 	MG----WMENANHKIAASAVGRWFQLDGSGH0PRERKGSLFFTEIRAGLATFFAMA~YII
AN8534.1  	MG-YAEWIGRTNAAVARSPVGRWFRLEGSGH0PRERKGAYFFTELRAGLATFFAMA~YII
          	**  ..*:   :  :. * ** **:*:****  :****: * **:**********: ***

MG08279.1 	AVNASIVS~DTGGTCTC--DRTIDPTCVADQAYALCKSEIRRDMITATAAISALGSFFMG
NCU01882.1	AVNSSVVS~DTGGTCVCTADPNVDRWCIDDPTYAICKAEVKRDLITATAAIAAFATFFMG
FG11617.1 	AVNSSIVS~ESGGTCVCPAYK--DGACVPDDAYQLCVAEVKRDAVTATAAISALATFFMG
AN8534.1  	SVNANITS2DTGGTCVCPAED-LATACDNNTEYLLCKQEVNRDLVTATAAIAAIASFFLG
          	:**:.:.* ::****.*.:       *  :  * :*  *:.** :******:*:.:**:*

MG08279.1 	LLANM~PVGIAPGMGMNA2YFAYTVVGFNGTGLVPYQVAVTAIFVEGFIFFGLALLGMRQ
NCU01882.1	LLANL2PIGLAPGMGLNA~YFAYTVVGFHGQGLVPYQVAVTAIFVEGWVFFGLALLGMRQ
FG11617.1 	LFANL~PVGLAPGMGLNA~YFTYTVVGPGGSGPVPYELALTAIFIEGFIFFGLALFGMRQ
AN8534.1  	LLANL~PVALAPGMGLNA~YFAYTVVGHHGTGLIPYSLAVTAVFVEGWIFLGLTLLGIRQ
          	*:**: *:.:*****:** **:*****  * * :**.:*:**:*:**::*:**:*:*:**

MG08279.1 	WLARAIPRCIKLATSVGIGLFLTIIGLTYSQGIGLIVGSVSTPLELAGCAPEDRITREGP
NCU01882.1	WLARVIPRSIKLATSVGIGFFLTLIGMTYSEGIGLVVGDTSVPLDLAGCHPSSRDSVTG-
FG11617.1 	WLARAIPRCIKLATSVGIGLFLTLIGLTYSEGIGLIVGSTSTPLELAGCEASYRDPATG-
AN8534.1  	WLARAIPASIKLATGAGIGLYLTLIGLSYSAGLGVVQGGTSSPIQLAGCAS-DTFGDDG-
          	****.** .*****..***::**:**::** *:*:: *..* *::**** .       * 

MG08279.1 	GGTEVLGCPGSHKMRNPALWVAIFCGGVFTVILMMYRVKGAIIAGILLVSIISWPRGTDL
NCU01882.1	------ACPDSDKMRNPTMWIGIFCGGVLTTVLMMYRVKGAIIAGIILISIISWPRTTEV
FG11617.1 	------LCPSSQKMRNPSMWIGIFCGGILTVLLMMYRVKGAVIAGILLVSIISWPRGTDV
AN8534.1  	------LCPSSEKMRNPTMWIGIFCGGVFTVFLMMYRVKGAVIAGILLVSIISWPRPTPV
          	       **.*.*****::*:.*****::*..*********:****:*:******* * :

MG08279.1 	TYFPYTPVGDDNFDFFRRVADFHPISRTLAVQEWNIGNYGGQFGLALITFL~YVDILDCT
NCU01882.1	TYFPYTAVGDDAFDFFKKVVDFHQIKHVLNVQQWDISGHGGQFGLALITFL~YVDILDTT
FG11617.1 	TYFPYDTLGDDRFDFFKKVVDFHQIKRTLNVLQFDISGHSGQFGLALITFL~YVDILDCT
AN8534.1  	TYFPHTETGDSSFDFFKKVVTFHPIQHTLVAQEWNISSNGGQFGLALITFL0YVDILDAT
          	****:   **. ****::*. ** *.:.* . :::*.. .*********** ****** *

MG08279.1 	GTLYAMAKHADLMDPVTQDFEGSTIA~Y~MVDSIAISIGALFGTPPVTAFVESGAGISEG
NCU01882.1	GTMYAMARYASLVDEETGDFEGSTIA2Y~MVDSVSIAIGAILGTPPVTAFVESGAGIGEG
FG11617.1 	GTLYGMARFADLVDPVTQDFEGSSIA~Y~MVDALSISIGAVLGVPPVTAFVESGAGISEG
AN8534.1  	GTLYSMAKFAGAMDERTQDFEGSAMA~Y0MVDAICISIGSLFGSPPVTAFVESGAGISEG
          	**:*.**:.*. :*  * *****::* * ***::.*:**:::* *************.**

MG08279.1 	GKTGLTAMTTGLCFFISIFFAPIFASIPSWATGCVLILVGSMMVRNVTEINWNYMGDAVP
NCU01882.1	GKTGLTAMATGVCFFIAIFFAPIFASIPPWATGCVLILVGSMMVRAVTDINWKYMGDAIP
FG11617.1 	GKTGITAMVAGICFFISIFFAPIFASIPPWATGCVLILVGSMMVGAVTEINWKYMGDAVP
AN8534.1  	GKTGLTSCMTGICFFIAVFFAPIFASIPPWATGSTLVIVGSMMMHATLEINWRYLGDAVP
          	****:*:  :*:****::**********.****..*::*****:  . :***.*:***:*

MG08279.1 	AFVTIALMPFTYSIADGLIAGICLYILINTLVWAIEKASGGRLVPHNKES-KDPWTWRIP
NCU01882.1	AFVCIALMAFTYSIANGLIGGICLYMLLNVLVWIIEKVSGGRIVPQNKEF-KDPWVPDF-
FG11617.1 	AFLTIAIMPFAYSIADGLIAGICTYMVLNTVVLLVKIISGGRIVPPNYEE-RDGWTWRIP
AN8534.1  	AFLTISLMPFTYSIADGLIAGILSYILINGGVWIVAKLTGGRISPPNREEEHEPWTWKIP
          	**: *::*.*:****:***.**  *:::*  *  :   :***: * * * .:: *.  :.

MG08279.1 	GGVLPQWMVRLSRGKKDFWRGDRPVQGEGASDGDEPRPGPGAVALIRDDADHPMGEKRVS
NCU01882.1	GSIIPMWMVNVAKG-RAPWKMDEPVRSIEGTTGPEP----------------VLVGEKGE
FG11617.1 	GGFLPPWMNRLAHGKKDFWREDNPP----HNTNNET----------------VVSDTAAP
AN8534.1  	GGFFPPWLVRAVHGKKDFWRAEDDAN--------------------------DLSLGVMP
          	*..:* *: .  :*.:  *: :                               :      

MG08279.1 	NGDSPERRGSSDVVAAATPPVQTSGDKKLM
NCU01882.1	KGDSSAGSRPDSATEVPAELVNEKGAHH--
FG11617.1 	KENGSDHGGSEGGKVAETTLPREKET----
AN8534.1  	HGSLSSQDRAGEKLASSKEQVTEK------
          	: . .    .             .
```
